# Dynamic Digital Radiography Pulmonary Function Testing: A Machine Learning Lung Study Alternative

**DOI:** 10.1016/j.chpulm.2024.100052

**Published:** 2024-03-29

**Authors:** Valeria Santibanez, Thomas J. Pisano, Florence X. Doo, Mary Salvatore, Maria Padilla, Norma Braun, Jose Concepcion, Mary M. O'Sullivan

**Affiliations:** a Department of Pulmonary, The Mount Sinai Hospital, New York, NY; b Department of Neurology, University of Pennsylvania, Philadelphia, PA; c Department of Radiology, Stanford University Healthcare, Palo Alto, CA; d Department of Radiology, Columbia University Irving Medical Center, New York, NY; e Department of Pulmonary, Mount Sinai Morningside, New York, NY; f Department of Radiology, Mount Sinai Morningside, New York, NY

**Keywords:** convolutional neural network, diaphragm physiology, dynamic digital radiography, lung areas, machine learning, pulmonary function testing

## Abstract

**Background:**

Common diagnostic tests for pulmonary disorders include chest radiography and pulmonary function tests (PFTs). Although essential, these tests only offer a static assessment. Chest dynamic digital radiography (DDR) integrates lung and diaphragm motion in one study with limited radiation exposure. DDR is relatively easy to obtain, but barriers to its clinical adoption include time-consuming manual analysis and unclear correlation with PFTs.

**Research Question:**

Can a machine learning pipeline automate DDR analysis? What is the strength of the relationship between PFT measures and automated DDR-based lung area measurements?

**Study Design and Methods:**

PFT and DDR studies were obtained in 55 participants. We developed an analysis pipeline using convolutional neural networks capable of quantifying lung areas in DDR images to generate DDR-based PFTs (dPFTs). PFT and dPFT measures were correlated in patients with normal, obstructive, and restrictive lung physiology.

**Results:**

We observed statistically significant (*P* < 1 × 10^-6^), strong correlations between dPFT areas and PFT volumes, including total lung capacity (*r* = 0.764), FEV_1_ (*r* = 0.591), vital capacity (*r* = 0.763), and functional residual capacity (*r* = 0.756). Automated DDR and lung shape tracking revealed differences between normal, restrictive, and obstructive physiology using diaphragm curvature indices and strain analysis measurements. Linear regressions allowed for derivation of PFT values from dPFT measurements.

**Interpretation:**

Statistically significant correlations found between dPFTs and PFTs suggest that dPFTs can act as a surrogate to PFTs when these are not available or unable to be performed. This study contributes to the potential integration of DDR as a reliable alternative to PFTs.

**Take-home Points**

**Study Question:** Is an automated machine learning analysis dynamic digital radiography analysis pipeline using convolutional neural networks capable of quantifying lung areas to create dynamic digital radiography-based pulmonary function tests (dPFTs)?

**Results:** dPFT measures strongly correlate with analogous pulmonary function test measures, suggesting dPFTs can act as a pulmonary function test surrogate.

**Interpretation:** The dPFT pipeline enables unique analyses including quantifying diaphragm curvature changes and lung-strain analysis. In a small cohort, dPFTs are capable of separating patients with normal lung physiology from patients with abnormal lung physiology (obstructive and restrictive diseases).

Mainstays of pulmonary imaging (eg, chest radiography [CXR], chest CT scan) provide static assessments of the dynamic respiratory cycle. Pulmonary function tests (PFTs) provide crucial physiologic data but lack anatomic information and can be burdensome to acquire.^1^, ^2^, ^3^ During disease exacerbations, PFTs are often impractical, making the understanding of natural disease history incomplete.

Chest dynamic digital radiography (DDR) can capture active lung movement during respiration by sequential radiography, offering a detailed dynamic view of lung and diaphragm motion. DDR is easy to obtain, requiring patients to breathe in front of a detector to capture the complete inspiratory and expiratory cycle, with radiation exposure comparable to static two-view CXR, or low-dose chest CT scan.^4^, ^5^, ^6^, ^7^, ^8^

Manual DDR-image analysis is time-consuming. We developed an automated analysis pipeline capable of calculating DDR-based projected lung areas (PLAs). PLAs in traditional chest radiographs correlate with total lung capacity (TLC).^9^, ^10^, ^11^ Previous studies suggest a clinical role for DDR in lung function evaluation,^12^^,^^13^ demonstrating PLA correlation with vital capacity (VC) in healthy adults.^14^ In mild interstitial lung disease, PLAs correlate with FVC, TLC, and FEV_1_ % predicted.^15^ Finally, PLAs and FVC correlate in patients with cystic fibrosis.^16^

If robust correlations exist, DDR could act as surrogate for PFTs; thus, we sought to further evaluate the correlations between PFT volumes (eg, TLC) and DDR-based PFT (dPFT) areas (eg, total lung area). To accomplish this, we created an analysis pipeline using convolutional neural networks (CNNs) to quantify lung areas in DDR image sequences during normal respiration, maximum inhalations, and exhalations. We compared PFT with dPFT measures in patients with normal and abnormal PFTs, observing strong correlations between the two methodologies. dPFT-based strain analysis further quantitatively captures known physiology of limited lung wall movement of COPD hyperinflation and restrictive conditions, further highlighting the capability of dPFTs.

## Study Design and Methods

The study was approved by Mount Sinai School of Medicine’s Institutional Review Board (FWA No. 00005656-5651, IRB STUDY-17-00676-MOD001).

Patients were recruited during outpatient pulmonary appointments from two hospitals. Exclusion criteria included the following: < 18 years of age, pregnancy, lactating individuals, and those who could not independently consent. Patients unable to undergo PFTs and DDR within a 150-day period were excluded. There were no disease exacerbations, changes in management, or diagnoses during this period.

### Data Acquisition and Preprocessing

During DDR, patients followed a breathing protocol of two tidal respirations, one maximal inspiration and expiration, and then tidal breathing. One respiratory cycle was defined from a resting end-expiratory position to the next resting end-expiratory position. Our protocol most closely approximates slow VC acquisition rather than FVC. The ratio of slow VC over FEV_1_ has been suggested to be more sensitive in detecting obstructive lung disease.^17^ PFTs were obtained by American Thoracic Society standards,^18^^,^^19^ measuring TLC, VC, FEV_1_ % predicted, tidal volume, inspiratory capacity (IC), and functional residual capacity (FRC).

**Figure 1:**
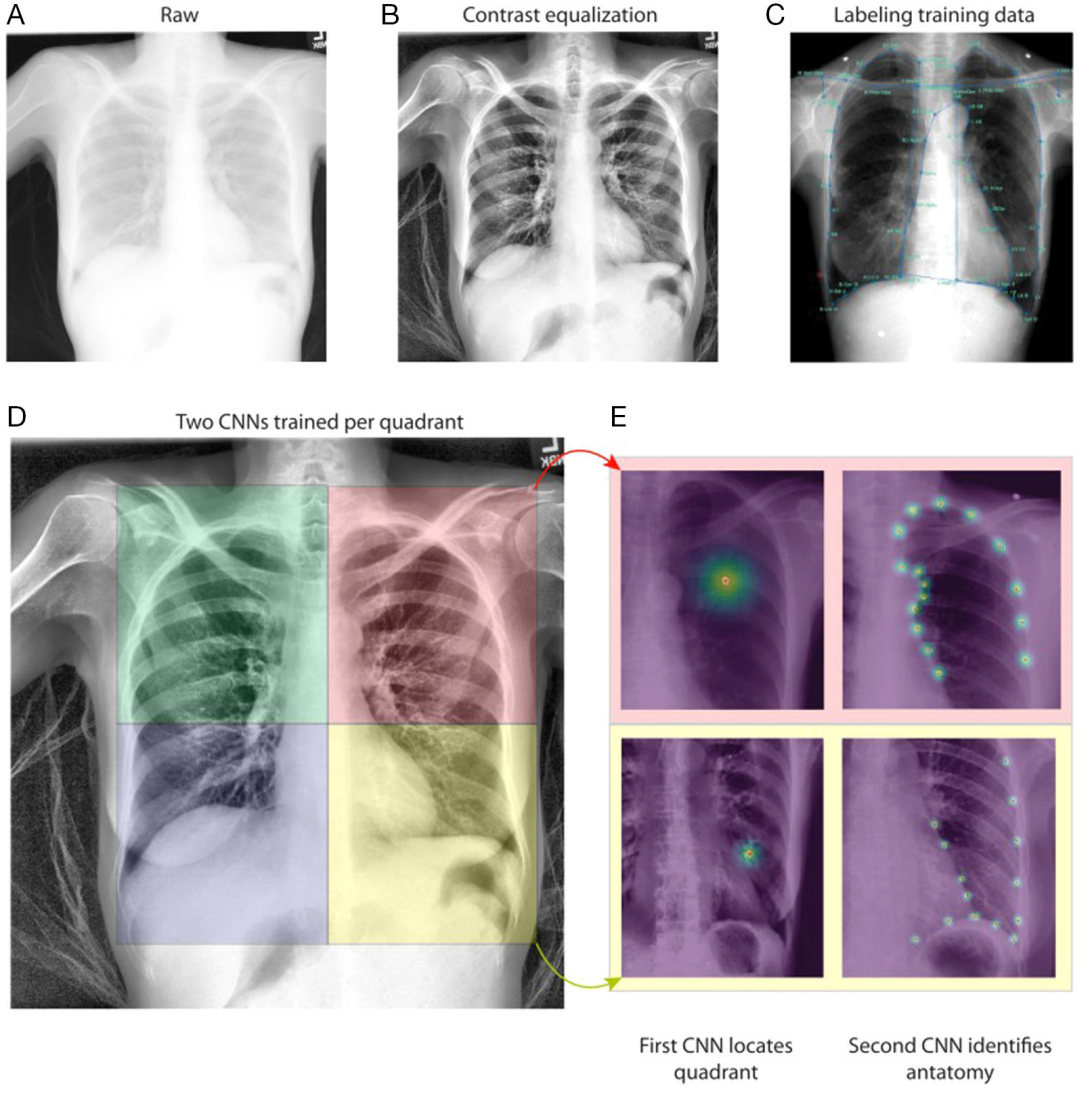
A-E, An automated pipeline for anatomic delineation of thoracic radiography using CNNs. A, Raw example of a dynamic digital radiograph. B, Contrast equalization using contrast limited adaptive equalization shows improved anatomic visualization. C, Training data are generated through manually labeling using Social LEAP Estimates Animal Poses’s graphical user interface. D, E, Neural network detection schematic. For each quadrant a series of two networks were used. The first identifies the quadrant center. The second, using input of the first, identifies individual anatomic landmarks within a quadrant. D, Four neural networks were trained, each with the objective of identifying a chest quadrant centroid. The four separate quadrants are visualized as colored boxes (right upper quadrant, green; right lower quadrant, blue; left upper quadrant, red; left lower quadrant, yellow). E, Serial centroid then anatomic identification. Two quadrant examples showing confidence maps of trained neural networks identifying quadrant centroids and then anatomic confidence maps. CNN = convolutional neural network.

DDR sequences acquired using commercially available DDR machines (Konica Minolta Healthcare Americas) used exposure conditions similar to published studies.^12^^,^^14^^,^^20^^,^^21^ Detector pixel size was 400 × 400 μm, captured at 15 frames per second, via pulsed x-ray at 15-Hz images with comparable pixel resolution to CXR. DDR total dose was estimated at 0.3 to 1.0 mGy when including posteroanterior (PA) and lateral views. PA views were analyzed for this study. Encrypted data were accessible only to research personnel. Preprocessing used a contrast adaptive local histogram equalization filter (CLAHE; cv2.create CLAHE, clip limit 8, tile grid size of 10 × 10 pixels) (Fig 1A, 1B). CNN training used Social LEAP Estimates Animal Poses, an object tracking software.^22^ After preprocessing, images were labeled with 61 points. Optimal pipeline performance was achieved with Social LEAP Estimates Animal Poses’s top-down approach in each of four lung quadrants (Fig 1). In each quadrant, the first neural network identified the quadrant center, followed by a second neural network that labeled anatomic points. Our approach totaled eight CNNs. Other training schemas were tested; however, we noticed improved performance when a neural network identified approximately 15 rather than 61 points. To enhance performance, we iteratively added new labeled frames to the training set, including images underrepresented in the data set (eg, maximal inspiration), allowing targeted training data set improvement.

After each training iteration, manual curation by two users separated tracked frames into acceptable or unacceptable tracking. Frames with unacceptable tracking were labeled and included in the subsequent training. Sixteen DDRs of the original 99 were excluded because both curators deemed CNN tracking unacceptable (see e-Table 1). Anecdotally, poor tracking was mostly related to x-ray underpenetration in patients with a BMI > 40 kg/m^2^.

### CNN Inference and Data Quantification

The final CNN generated anatomic point coordinates for each frame. Custom Python software calculated areas by summing pixels within polygons for each structure (cv2.contourArea) (see e-Table 2 for structure-specific points). Curvature indices, obtained from the diaphragm’s short (vertical) to long (horizontal) radius ratio, are described in e-Figure 5. In cases of missing points in a frame (occurring when CNN probability maps for diaphragm points fell below CNN prediction threshold), point location was inferred using adjacent points. After Gaussian smoothing (scipy.ndimage.gaussian_filter1d, kernel size 3), local minimum and maximum lung areas across 15 preceding and succeeding frames were quantified (scipy.signal.argrelextrema, order = 15). Peaks and troughs identified initiation of exhalations and inhalations, respectively.

Flow was calculated by quantifying framewise area changes after Gaussian smoothing. Largest breaths were defined by maximal area. Pearson regressions are shown in Figure 4 (scipy.stats.pearsonr). Polynomial regressions with 1 and 3 *df* are shown in e-Figure 2 (np.poly1d, np.polyfit, sklearn.r2_score).

**Figure 2:**
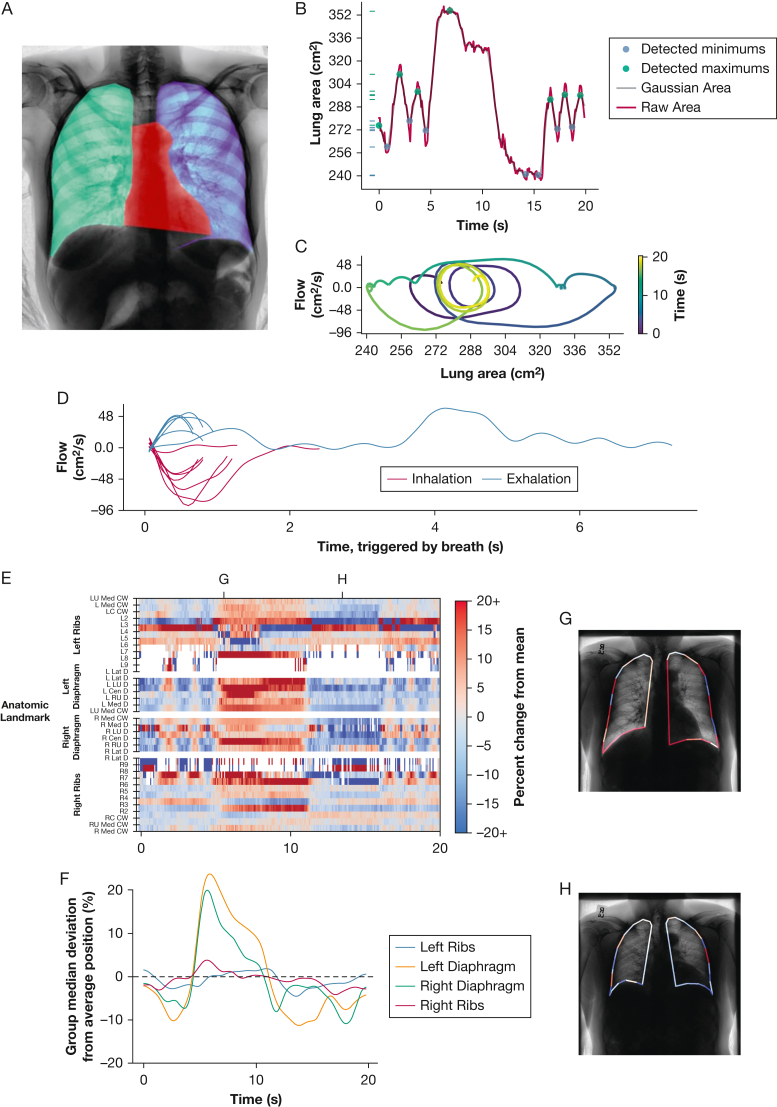
A-H, Dynamic digital radiography (DDR) can be used to quantify breathing dynamics and generate analogous pulmonary function testing measures. A, Lung areas are measured using DDR. Example images of right lung (green), left lung (purple), and cardiac (red) silhouettes are delineated using the automated pipeline. B, Changes in lung area over time (analogous to the pulmonary function test [PFT] change in volume over time) are plotted for a single DDR sequence of 300 frames. Red line depicts raw area measure, and gray line depicts area after Gaussian smoothing (3 kernel size). Green and blue dots correspond to automatically detected maximum and minimum values, respectively. C, Flow (change in area per second) vs lung area (measure analogous to PFT flow-volume loops). Line color delineates time. D, Flow by triggered breaths (time zero denotes detected minima/maxima displayed in A). Positive flow (blue) denotes exhalation, and negative flow (red) denotes inhalation. E, F, Point strain can be used to measure chest wall expansion during the breathing cycle. E, Point strain over time. Strain is defined as percent change from average distance between two points. G, H, Example inhalation (G) and exhalation (H) strain images. F, Median strain of the four functional groups (ribs and diaphragms of each side).

**Figure 3:**
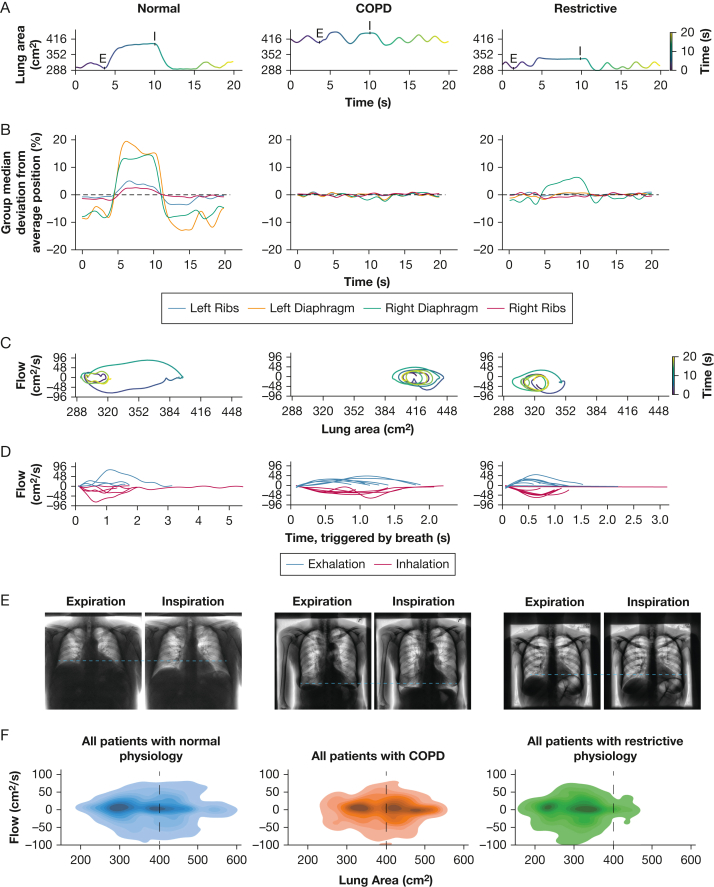
A-F, Dynamic digital radiography can detect known differences in obstructive and restrictive pulmonary physiology. Columns depict normal, obstructive, and restrictive examples. A, Change in lung area over time for each condition. B, Median strain of the four functional groups (ribs and diaphragms of each side). Note the patient with restrictive physiology has left-sided partial diaphragmatic paralysis. C, Flow-area loops highlight differences (normal), or lack thereof (obstructive/restrictive), between normal and deep breaths. Note the ability of the patient with normal physiology to expand their lungs during deep breathing relative to the other conditions (A-C). D, Flow by triggered breaths. E, Example expiration and inspiration images for each patient shown (corresponding frames are marked in A). F, Flow vs lung area kernel density estimations of all breaths for each pulmonary category (all breaths shown). Vertical lines at 400 cm^2^ highlight differences between lung area of maximal inspiratory effort (total colored area) and normal tidal volume areas (regions of increased density). Note the relative leftward shift of the density center for patients with normal and restrictive physiology, but patients with normal physiology are able to expand to larger areas during their maximum inspiration.

**Supplementary Figure 1:**
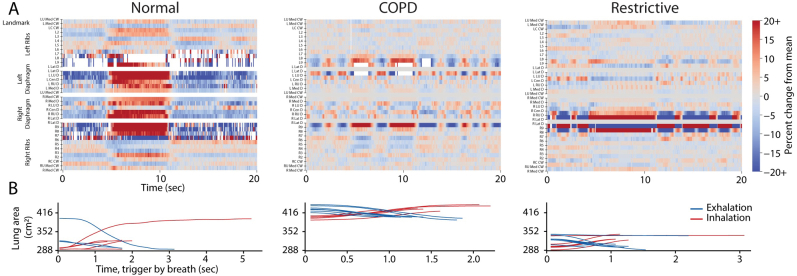


Borrowing a concept from the strain echocardiography field, we generated strain plots from anatomic point-pairs (Figure 2, Figure 3, Figure 5; e-Fig 1). Distances between point-pairs were measured (see e-Table 3 for list of pairs), and mean point-distances across a single study were determined. Percent change from mean was defined as follows: −100 × (1 − point-distance/mean point-distance); thus, positive percent change denotes inhalation (expansion of space between points) and negative percent change denotes exhalation (contraction of space). Group framewise median percent change was used for anatomically functional grouping (see e-Table 3 for grouping).

Similar to prior computational analysis pipelines,^23^ dPFT pipelines were run using custom Python 3+ code. Data frame manipulations used Numpy 1.14.3^24^ and Pandas 0.23.0.^25^ Plotting used Matplotlib 2.2.2^26^ and Seaborn 0.9.0.^27^ Image analysis and visualization used Scikit-Image 0.13.1,^28^ ImageIO 2.25,^29^ and SimpleITK 1.0.0.^30^ For statistical analyses, SciPy 1.1.0^31^ was used. Plotting and analysis used Seaborn 0.9.0^27^ and Scikit-Learn 0.19.1.^32^ Figures were exported from Python and modified for production using Adobe Illustrator (version 26.5) (Adobe). Our dPFT software can be found at https://github.com/tjp7rr/dPFT.

## Results

Ninety-nine patients underwent DDR. Fourteen lacked complete PFTs and 16 had poor DDR quality related to underpenetration, which was uncorrectable digitally, leading to exclusion. Eleven did not meet 150-day criteria between DDR and PFTs (e-Table 1), and three were excluded given suboptimal PFTs. Thus, 55 cases were included in the final analysis. Lung volume measurements were obtained by whole-body plethysmography in 82% (n = 45) and gas dilution (nitrogen washout) in 18% (n = 10). Power calculations (ɑ = 0.05, β = 0.2) for demonstrated PLA correlations^14^ suggested sample size requirements of 16 for VC (*r* = .65) and 25 for FEV_1_ (*r* = 0.54).

### dPFT Loops Quantify Respiratory Dynamics

After Gaussian smoothing of each point across adjacent frames, left lung, right lung, and heart areas were calculated (Fig 2A, e-Table 2), producing lung area-time plots (Fig 2B). These dPFT plots are comparable with PFT volume-time plots. Next, area-flow plots (area change per s) (Fig 2C) were generated, analogous to PFT flow-volume loops. Peak detection tracked inhalation-peaks and exhalation-troughs, generating triggered-flow plots (Fig 2D) and triggered-lung-area plots (e-Fig 1B). Unlike PFTs, DDR captures anatomy, enabling strain analysis (Fig 2E, e-Fig 1A), comparable with strain echocardiography.^33^ Strain plots represent framewise percent change from the average distance between points depicting relative chest expansion and contraction during respiration (Figs 2G and 2H are representative frames marked on Fig 2E). To efficiently visualize chest wall (ribs) and diaphragm movement, average strain point-pairs were plotted (Fig 2F; e-Table 3 details strain point-pairs).

Physiologic differences^34^ can be observed using dPFTs, exemplified by three cases: normal, COPD, and restrictive disease (Fig 3). Lung area-time (Fig 3A) demonstrated distinctive dynamic area ranges during peak inhalation and exhalation. Flow-area loops (Fig 3C) showed higher values in COPD, consistent with hyperinflation, and diminished volumes in the restrictive case.^35^ Strain (e-Fig 1A) and average grouped strain plots (Fig 3B) showed greater dynamic range for normal relative to COPD and restrictive examples. The patient with restrictive disease was selected given known left hemidiaphragmatic paresis, which can be observed using strain analyses, further highlighting the utility of this approach ( Fig 3B, e-Fig 1A). Breath-triggered flow (Fig 3D) showed faster area changes for normal relative to COPD and restrictive examples. Physiology patterns are illustrated with inspiratory and expiratory images (Fig 3E). Flow-area density plots demonstrated class differences (Fig 3F). Leftward density shift and decreased lung area is seen in restriction, suggesting smaller lung areas and dynamic range. COPD physiology demonstrated a rightward shift in lung area density, likely from COPD hyperinflation, and decreased lung area dynamic ranges when compared with normal physiology.

### dPFTs Strongly Correlate With PFTs

**Figure 4:**
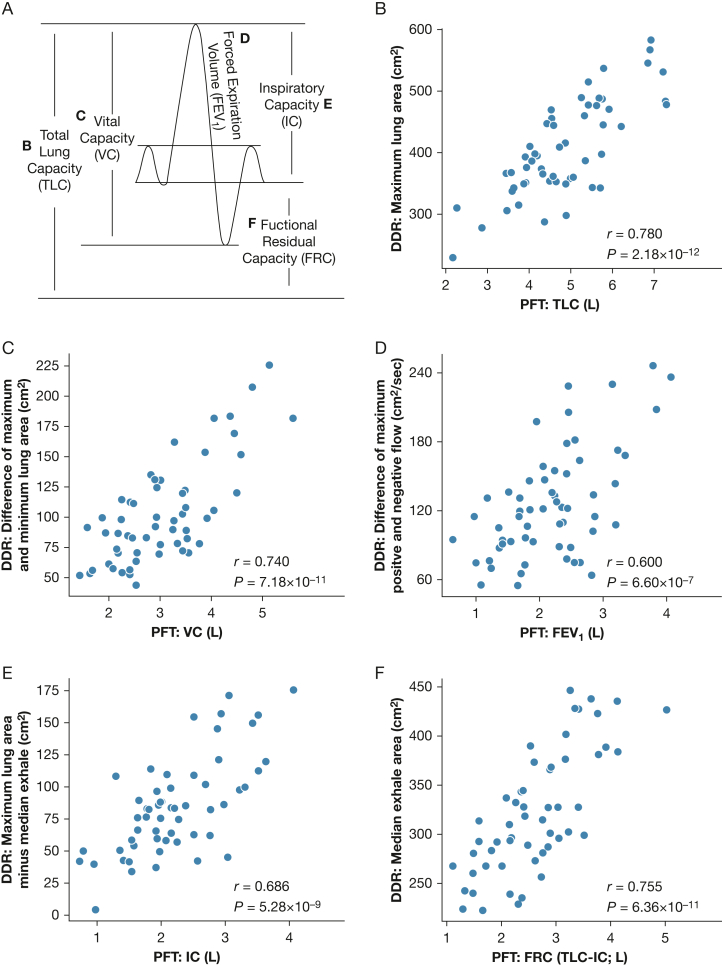
A-F, DDR measures strongly correlate with pulmonary function measures. A, Schematic showing PFT correlates present in the remainder of the figure. B, TLC (PFT) vs maximum lung area (DDR). C, VC (PFT) vs maximum-from-minimum lung area difference (DDR). D, FEV_1_ (PFT) vs difference of maximum positive and negative flow (DDR). E, IC (PFT) vs difference of maximum lung area from median minimum value (DDR). F, Residual volume (PFT) vs minimum lung area (DDR). Pearson correlation *r* and significant (*P*) values are shown. DDR = dynamic digital radiography; FRC = functional residual capacity; IC = inspiratory capacity; PFT = pulmonary function test; TLC = total lung capacity; VC = vital capacity.

To determine dPFT reliability as surrogate for PFTs, we correlated PFT volume measures with analogous dPFT area measures (Fig 4A). Maximum lung areas strongly correlated with PFT TLC (Pearson correlation *r* = 0.764; *P* = 7.61 × 10^−13^) (Fig 4B). A VC surrogate, the difference between DDR maximum and minimum lung areas strongly correlated with PFT VC (*r* = 0.763; *P* = 3.53 × 10^−13^) (Fig 4C). PFT FEV_1_ with a 1-s DDR area change comparison was deferred, given no absolute start time for DDR maximum inhalation and exhalation (self-initiated by patient after tidal breaths). Instead, DDR-based flow change, the difference between absolute positive flow (marker of exhalation ability) and negative flow (marker of inhalation ability), strongly correlated with FEV_1_ (*r* = 0.591; *P* = 3.37 × 10^−7^) (Fig 4D). The median exhalation area (median minimum as depicted in Figure 2B) subtracted from the maximum lung area, a DDR-analogous IC measure, significantly correlated with PFT IC (*r* = 0.708; *P* = 8.92 × 10^−11^) (Fig 4E). Finally, PFT FRC strongly correlated with a DDR-analogous median exhalation area (*r* = 0.756; *P* = 1.96 × 10^−12^) (Fig 4F).

**Supplementary Figure 2:**
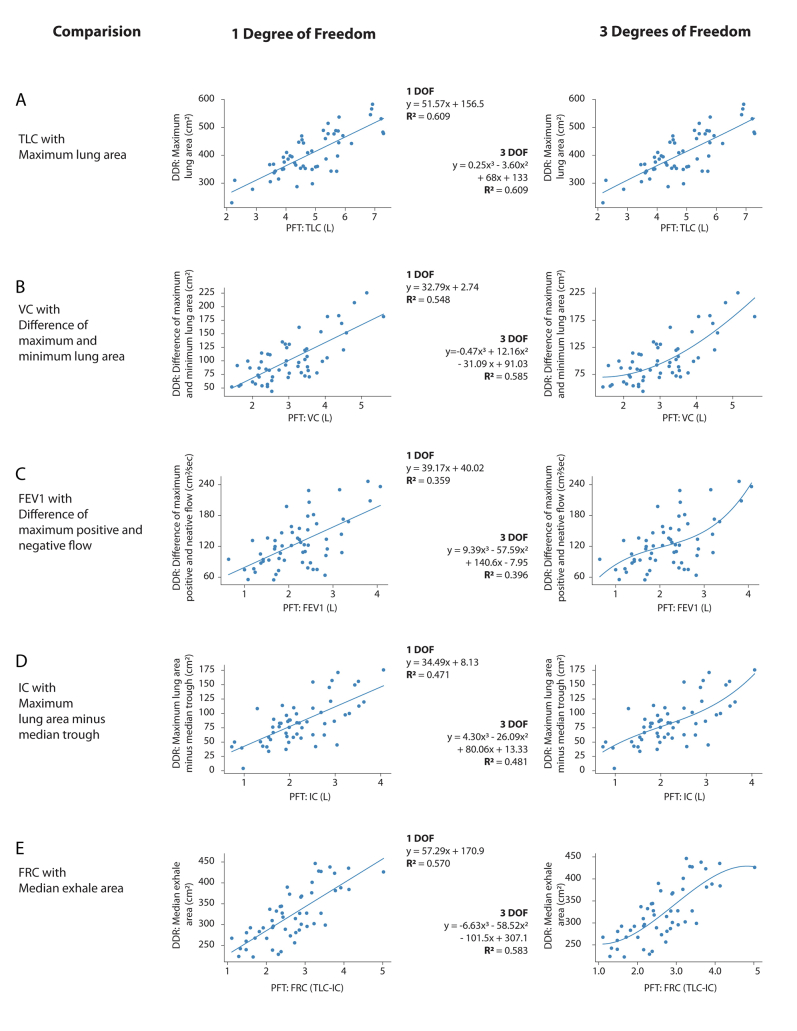


**Supplementary Figure 3:**
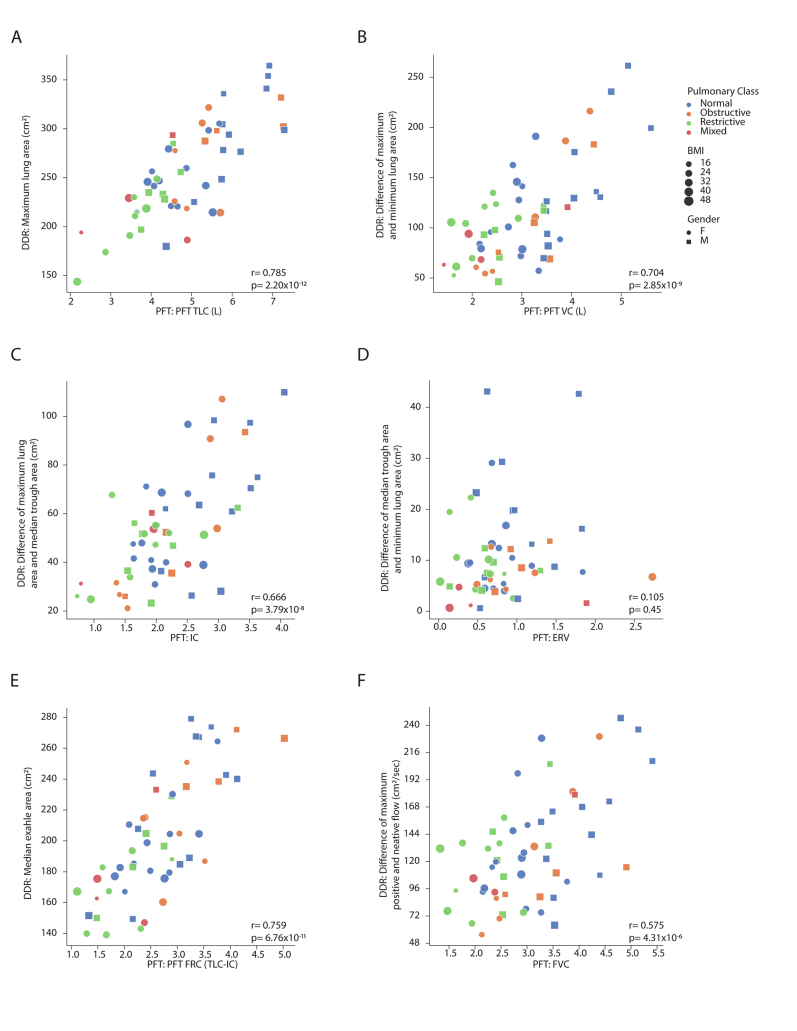


All five dPFT measures had a primarily linear relationship (e-Fig 2). Anatomy-related (e-Fig 3A) and flow-related (e-Fig 3B) plots by pulmonary condition, gender, and BMI are included. Besides underpenetration, suboptimal DDR tracking often involved the cardiac silhouette. To combat this, we tracked the entire lung area without subtracting out heart area (e-Fig 6); these correlations remained strong and predominantly linear, again suggesting that dPFTs might be reliable PFT surrogates.

### Strain Analysis Reveals Different Dynamics

**Figure 5:**
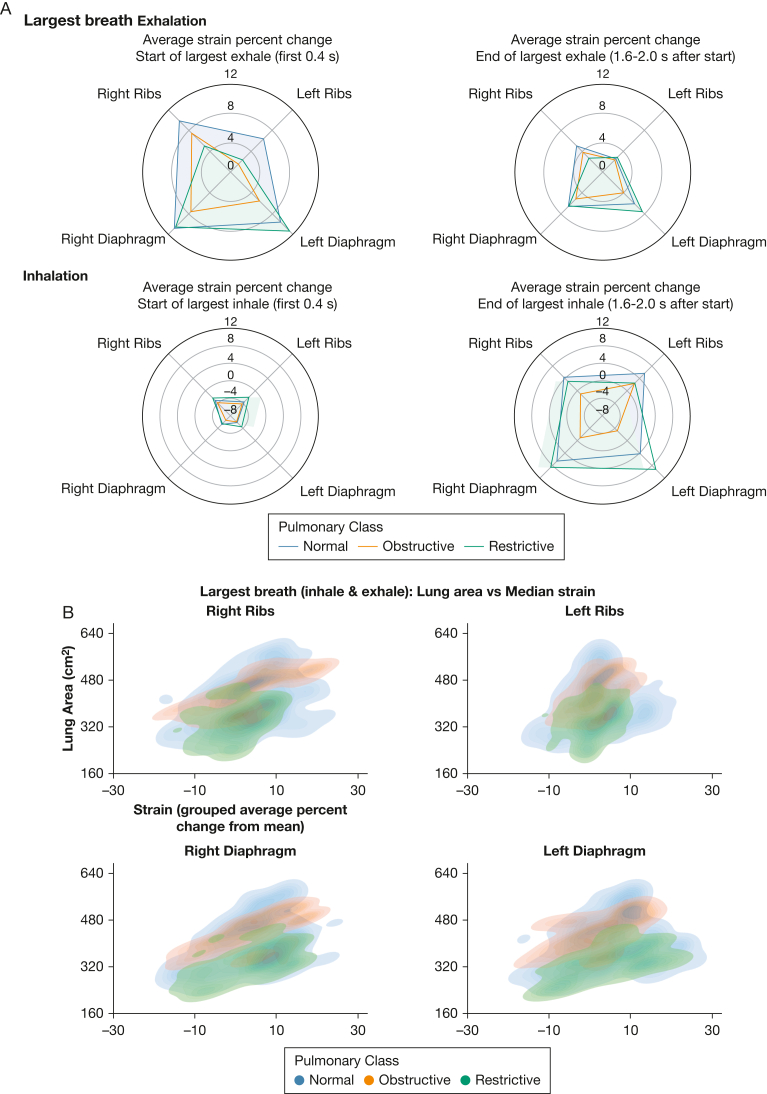
A, B, Grouped strain analysis by pulmonary class reveals different lung movement patterns. Plots are grouped by pulmonary class: normal (n = 26), COPD (n = 10), and restrictive (n = 15). A, Radar plots of grouped mean strain percent change of the largest breath by area for each pulmonary condition. Left column depicts the first six frame average (0.4 s) of the start of inhalation or exhalation, whereas the right column depicts the average of frames 24 through 30 (1.6-2 s) after initiation. B, Lung area vs median strain density plots of largest breath grouped by pulmonary class. Each plot shows one of the four functional groups (ribs and diaphragm of each side).

**Supplementary Figure 4:**
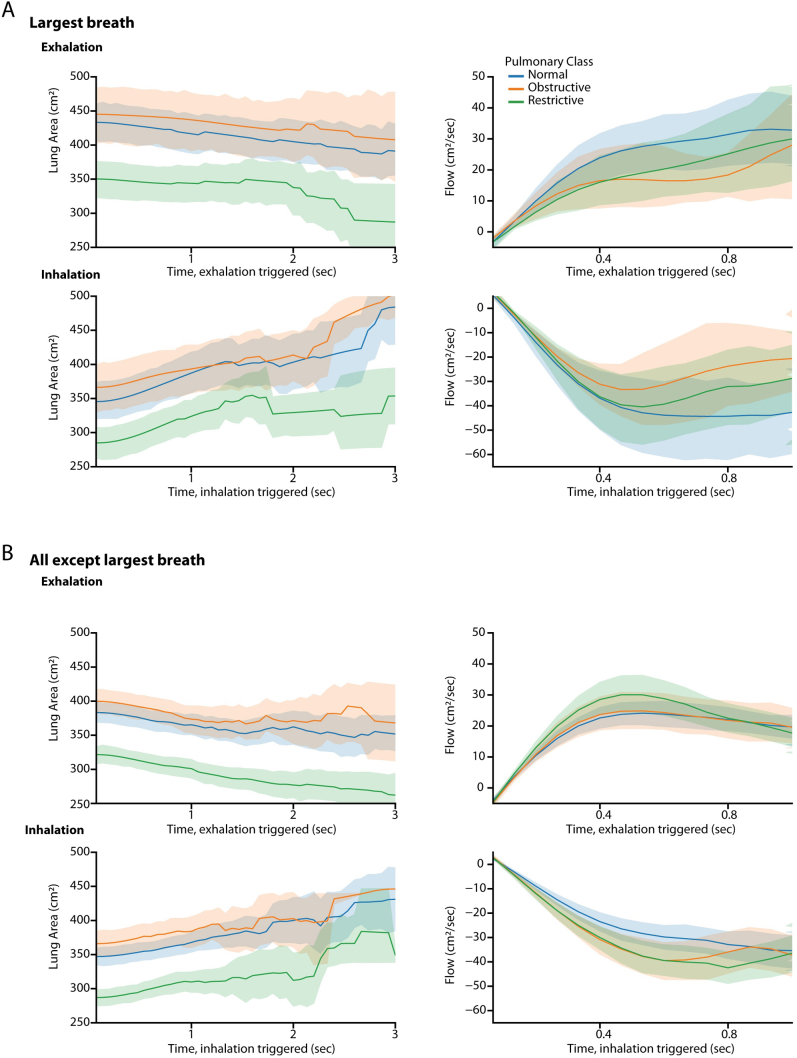


Radar strain plots grouped by physiologic condition identified respiratory dynamic differences of each patient’s largest exhalation and inhalation (Fig 5A). Interestingly, normal lung physiology had the most strain—relative lung tissue expansion—at the largest exhale start (Fig 5A). Restrictive physiology had almost comparable diaphragm strain, but reduced rib movement compared with patients with normal lung physiology, suggesting restrictive physiology may compensate for decreased wall movements by increasing diaphragm movement. At largest inhale initiation, COPD physiology had less lung contraction (Fig 5A). Decreased rib movement in restrictive disease and diminished exhalation in COPD align with expected pathophysiology. In exploring the lung area-to-regional strain relationship (Fig 5B), again patients with normal lung physiology exhibited the largest dynamic ranges for both lung area and strain. Restrictive physiology showed decreased dynamic ranges of bilateral rib cage (e-Fig 4). Strain analysis efficiently captures the breathing dynamics of various pulmonary physiologies.

### Curvature Index Enables Diaphragm’s Quantification

**Supplementary Figure 5:**
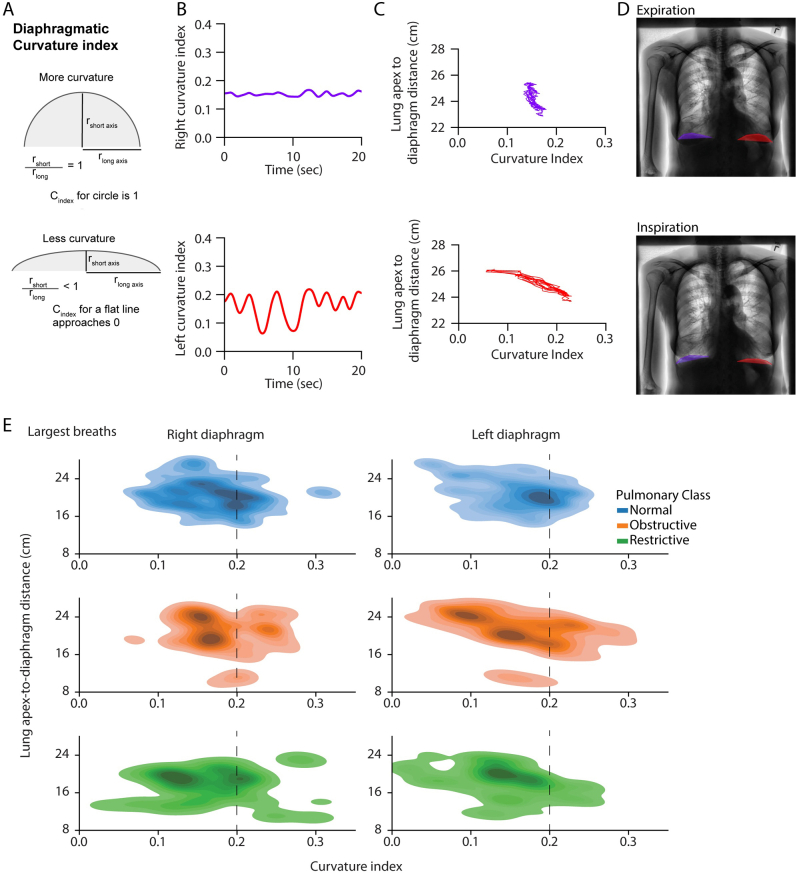


To compare diaphragm movement and curvature change across pulmonary conditions, we created a curvature index, defined as the vertical-to-horizontal diaphragm radius ratio (e-Figs 5A-D). Higher curvature index indicates more curved diaphragm (ie, normal diaphragm) relative to a more flattened diaphragm, often seen in COPD.^36^ Indeed, density plots of lung apex-to-diaphragm distance with curvature index (e-Fig 5E) show that both patients with COPD and with restrictive disease have lower curvature indices (flatter diaphragms). Curvature index offers an avenue for future study in progressive respiratory failure.

### Pulmonary Reserve to Separate Categories

Similar to breathing reserve,^37^^,^^38^ we sought to define dPFT measures to categorize lung conditions and quantify spare lung function. Toward this goal, we introduced DDR pulmonary reserve as follows:

$$\text{DDR}\;\text{pulmonary}\;\text{reserve}=\left(1\;-\;\frac{\text{DDR}\;\text{median}\;\text{tidal}\;\text{breath}\;\text{area}}{\text{DDR}\;\text{VC}}\right)\times 100$$

**Figure 6:**
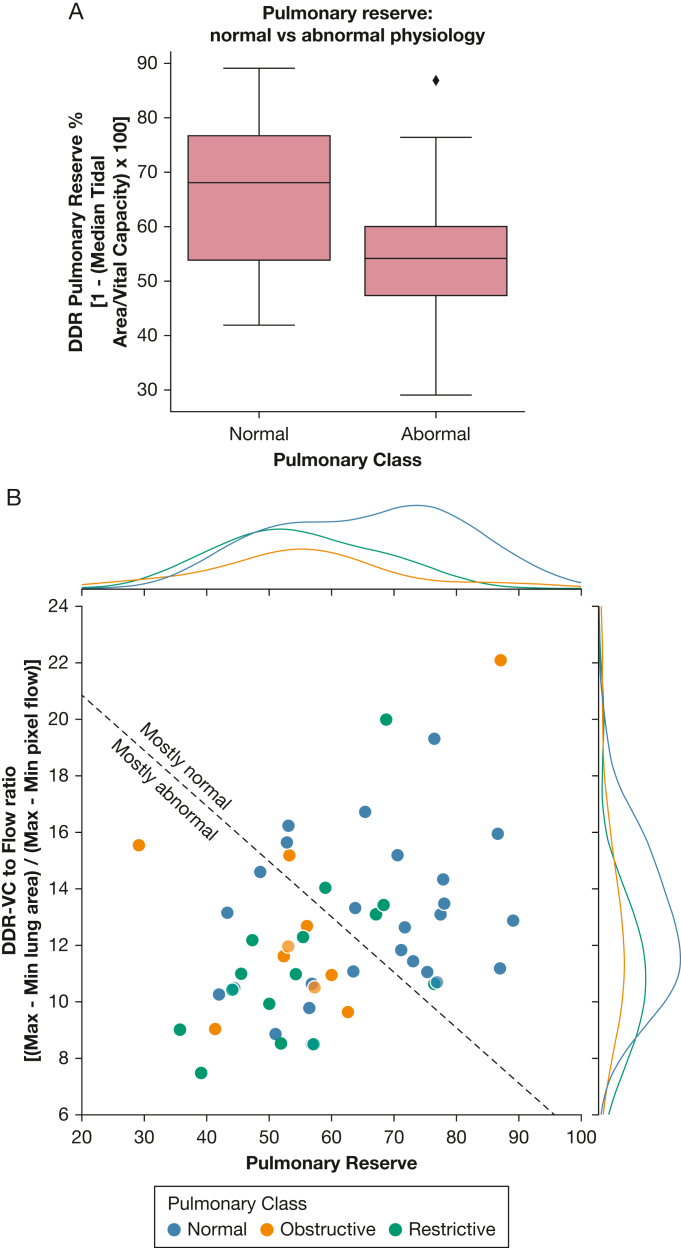
A, B, Pulmonary reserve is a helpful measure to separate pulmonary classes. A, Pulmonary reserve boxplots between normal and abnormal (COPD and restrictive) pulmonary classes. Pulmonary reserve is defined as 1 − (median tidal breath area/vital capacity) × 100. Generally, this number describes how close a person’s tidal breaths are to their VC. A smaller reserve capacity might suggest a patient is breathing close to their lung’s maximal VC. A one-tailed *t* test revealed a *t* statistic of 2.88 (*P* = .0029). B, DDR VC-flow ratio to pulmonary reserve percent. DDR VC-flow ratio is a measure that with larger values suggests larger deformation capacity of lungs relative to rate of change. Dotted line drawn to depict an approximate boundary that mostly separates normal physiology from abnormal physiology. Kernel density estimations are shown for pulmonary conditions for each of the two axes. DDR = dynamic digital radiography; VC = vital capacity.

This value reflects the percent of lung area used during tidal breathing relative to the maximum VC, representing spare lung capacity available during normal breathing. This is a lung size independent measure. The closer the tidal breath volume is to the VC, the lower the pulmonary reserve. This value could help categorize pulmonary physiology and be used to trend lung function changes over time. Both COPD and restrictive conditions exhibited lower pulmonary reserve values than patients with normal lung physiology (Fig 6). A one-tailed *t* test between patients with normal and nonnormal physiology revealed a *t* statistic of 2.88 (*P* = .0029). Analysis of variance revealed an F statistic of 4.07 (*P* = .23).

To further separate the three pulmonary conditions, we compared pulmonary reserve values with DDR-based VC-to-flow ratios (Fig 6B). VC-to-flow ratio was defined as follows:

$$\text{DDR}\;\text{VC}\;\text{to}\;\text{flow}\;\text{ratio}=\frac{\text{VC}}{\text{delta}\;\text{flow}}=\frac{\text{DDR}\;\text{maximum}\;-\;\text{minimum}\;\text{lung}\;\text{area}}{\text{DDR}\;\text{maximum}\;-\;\text{minimum}\;\text{flow}}$$

A higher VC-to-flow ratio suggests a larger lung area change capacity relative to the rate of change. Healthy lungs have large area changes and generate high flows exhibiting high pulmonary reserve and VC-to-flow ratio. COPD-affected lungs are hyperinflated, with limited dynamic area range, only temporarily generating high flows before airway collapse; this results in a relatively smaller numerator and VC-to-flow ratio. Lungs affected by restrictive conditions have decreased lung compliance generating short, high-intensity flow over a small dynamic lung area, resulting in the smallest VC-to-flow ratio from a small VC numerator and a normal-to-above normal flow denominator. These ratio differences suggest dPFT measures might be able to separate normal from abnormal physiology (Fig 6B).

## Discussion

DDR provides a rich opportunity to study dynamic lung functioning; however, manual analysis is time-consuming. The dPFT pipeline uniquely facilitates quantification and generation of DDR-based lung area measures: dPFTs, as alternative to PFTs. The strong correlations between PFTs and dPFTs support a meaningful DDR-to-PFT relationship (Fig 4). Correlation values approximated prior manually determined DDR-to-PFT correlations, being superior^14^ and inferior to others^15^; however, our approach is entirely automated, enhancing DDR’s potential for routine clinical integration. dPFT measures strongly correlated with key PFT values including TLC, VC, FEV_1_, IC, and FRC (Figs 4B-F). Linear relationships allow for deriving PFT parameters from dPFT measures using regression formulas (e-Fig 2).

DDR has advantages over traditional studies. Beyond limiting radiation exposure,^4^, ^5^, ^6^, ^7^, ^8^ DDR enables functional study, exposing the dynamic interactions between lungs, diaphragm, and heart^39^, ^40^, ^41^ across a breathing cycle. Although PFTs are vital for diagnosing and monitoring pulmonary disorders, they pose challenges in accessibility and interpretation.^42^ Our findings add to growing evidence suggesting DDR as a potential PFT surrogate. The dPFT pipeline facilitates tracking anatomy (Figure 1, Figure 2), enabling lung area-time and flow-area loops analogous to PFT volume-time and flow-volume loops (Figs 2B, 2C). Using dPFTs, we observed differences in dynamic lung area between normal and abnormal physiology (e-Fig 1B; Figs 3A, 3C, 3F).

Diaphragm motion and respiratory muscle synchrony play a role in pulmonary disorders^36^ and are readily visible with DDR.^12^ Our study goes beyond tracking vertical diaphragm displacement^20^ and introduces multipoint diaphragm tracking (e-Fig 5), enabling the study of diaphragm impact in the work of breathing.^43^, ^44^, ^45^, ^46^ Enhancing diaphragmatic dynamic quantification, we introduced the concept of curvature index, the diaphragm vertical-to-horizontal radius ratio (e-Figs 5A-D). Flatter diaphragms (eg, in COPD) exhibit smaller curvature indices relative to patients with normal lung physiology (e-Fig 5E).

Adopting a strain echocardiography concept,^33^ we functionally grouped anatomic points (ribs and diaphragms of each side) to assess average group strain during maximal inhalation and exhalation (Fig 5). Strain analysis quantitatively captures the known physiology of diminished lung wall movement of both restrictive and COPD-related hyperinflation.

Our study suggests the use of dPFTs in patients unable to complete PFTs, including neuromuscular conditions leading to poor mouthpiece seal and nonambulatory states.^2^^,^^3^ PFTs are effort-dependent, subject to nuanced interpretation, and may be impractical to perform during disease flares or exacerbations.^34^^,^^47^^,^^48^ Although our study is cross-sectional, the ease of dPFT acquisition increases feasibility for longitudinal studies during baseline and disease flares.^16^ With sufficient validation, changes in dPFTs from baseline, combined with DDR pulmonary reserve (Fig 6), could serve as potential markers for treatment escalation.

dPFTs could be a valuable screening tool for pulmonary physiology workup considering its potential to separate normal from abnormal pathology (Fig 6), especially given the advent of portable DDR machines. In emergencies (eg, COPD exacerbations), a portable DDR with the automated pipeline could enable determination of dPFT values including pulmonary reserve. Comparing dPFTs during exacerbation and nonexacerbation might improve treatment decisions in emergency settings.

### Limitations

**Supplementary Figure 6:**
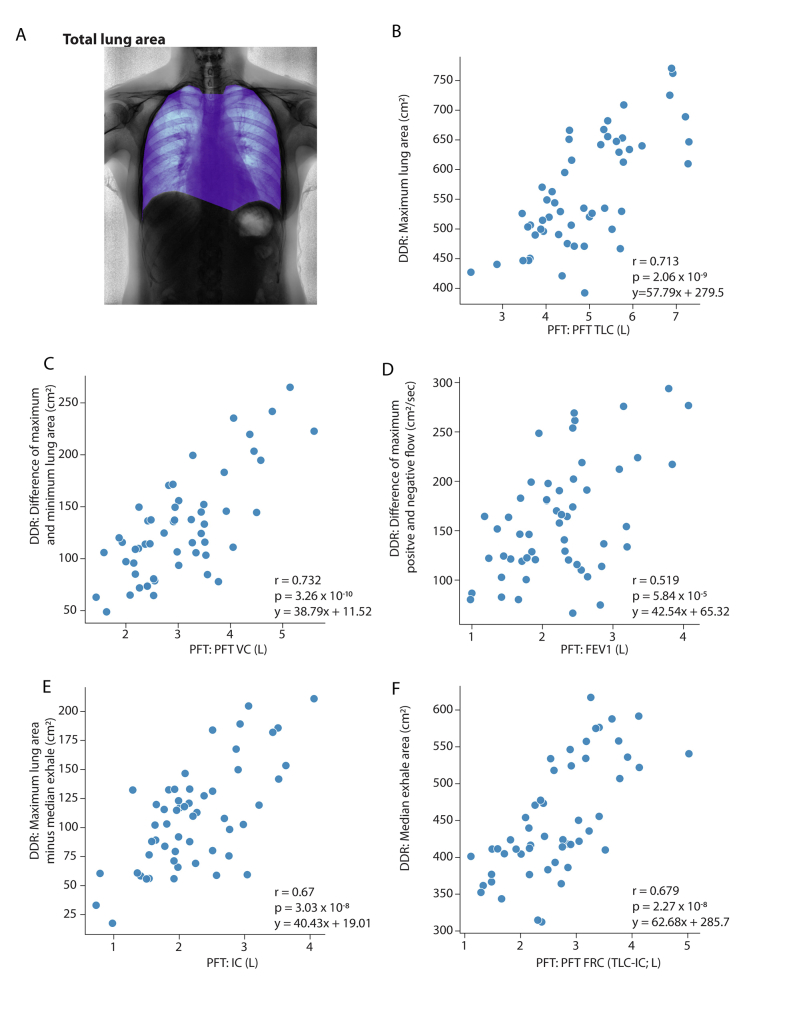


Limitations in generalizability of our study include recruitment from two hospitals and a small representation of patients with obstructive (n = 10) and restrictive (n = 15) physiology. Complex cases (eg, combined pulmonary fibrosis and emphysema) were not included. Separately, lung volume measurement methods varied among patients. Most underwent whole-body plethysmography (n = 45), whereas a minority underwent nitrogen washout (n = 10). Further studies are needed to understand dPFT diagnostic utility in complex conditions (eg, combined pulmonary fibrosis and emphysema) and the effects of different lung volume measurement methods on dPFT lung area correlations. Cardiac silhouette CNN tracking failures can be addressed by tracking entire lung areas (e-Fig 6). In future iterations, increasing the number of diaphragm tracking points might make the curvature index more robust. Unlike PFTs, breathing effort encouragement during DDR acquisition was passive, which might have negatively affected correlations and reliability. We did not use such thorough rehearsal procedures or prerecorded instructions as previously reported.^15^ Future standardization with recorded audio instructions might further improve correlations. Given the ability to acquire DDR in PA and lateral positions, future studies should incorporate both sequences into analysis for lung volumetric evaluation.

## Interpretation

Our study demonstrates robust dPFTs and PFTs correlations using an automated DDR analysis pipeline. This dPFT pipeline has potential to discern normal from abnormal physiology, suggesting dPFTs are valuable in assessing lung dynamics. Automated analysis enables both diaphragm-shape tracking and strain imaging, a novel deformation-based approach. Given the strong dPFT-to-PFT relationship, we advocate DDR use to measure pulmonary function when traditional PFTs are unavailable or unable to be performed particularly in emergency or inpatient hospital settings. DDR, with its easy acquisition, might be useful in quantitatively studying dynamic physiologic changes over time, and across disease flares, a study traditionally challenging with conventional PFTs.

## Funding/Support

The image device was provided by Konica Minolta.

## Financial/Nonfinancial Disclosures

None declared.

## Supplementary Data

e-Online Data
