# Supplementary material for: Dynamic Digital Radiography Pulmonary Function Testing: A Machine Learning Lung Study Alternative
Source: CHEST Pulm. 2024 Mar 29;2(3):100052. doi: 10.1016/j.chpulm.2024.100052 (PMC13418388; doi:10.1016/j.chpulm.2024.100052)
Supplement: e-Online Data [file mmc1.docx]

**Dynamic Digital Radiography Pulmonary Function Testing: A Machine Learning Lung Study Alternative**

**Supplementary Figure Legends**

**e-Figure 1. Strain maps and area-triggered breath plots show differences across pulmonary conditions.** (a) Strain maps for patients with PFT-defined normal (left), COPD (middle), and restrictive (right) lung conditions. Note the normal ability to expand their lungs during deep breathing relative to the other conditions. Also note the restrictive example has left sided partial diaphragmatic paralysis. (b) Area change by triggered breath highlights area differences (normal), or lack thereof (obstructive/restrictive), between normal and deep breaths. These differences between tidal areas and maximum areas reflect FRC and IRV. The same patient examples in Figure 2 are shown in this figure. Abbreviations: COPD, chronic obstructive pulmonary disease; FRC, functional residual capacity; IRV, inspiratory reserve volume; PFT, pulmonary function test.

**e-Figure 2. Pulmonary function testing correlations with dynamic digital radiography are mostly linear.** Polynomial regressions are shown for correlations between DDR and PFT measures with 1 degree of freedom (DOF; left side) and 3 DOF (right side). For most of the measures shown in (A-E) the R squared value only minimally improves when moving from 1 DOF to 3 DOF suggesting that a linear, 1 DOF, fit captures much of the relationship. Pearson’s correlations shown. Abbreviations: DOF, degree of freedom; DDR, dynamic digital radiography; PFT, pulmonary function test.

**e-Figure 3. PFT correlations with DDR correlations with pulmonary class.** Anatomy related and flow related correlations between PFT and DDR. Pulmonary classes are labeled by color, BMI by marker size and gender by marker. Pearson’s correlations shown. Abbreviations: DDR, dynamic digital radiography; PFT, pulmonary function test.

**e-Figure 4. Differences in breath dynamics of pulmonary classes.** (a) Largest breath lung area and flow changes for exhalation (top row) and inhalation (bottom) of normal (blue), obstructive (orange) and restrictive (green) groups. The single largest breath was defined as maximum lung area for lung area plots (left column) and maximum flow for flow plots (right column). Given the nature of breath instruction, almost always the same breath constituted both maximum area and flow. (b) Lung area and flow changes for exhalation and inhalation for all breaths except the largest categorized by pulmonary groups similar to A.

**e-Figure 5. Diaphragmatic curvature index is a promising method to quantify diaphragm dynamics** (a-d) Diaphragmatic curvature index example of patient with more dynamic left diaphragm relative to right. (a) Graphical representation of diaphragmatic calculation, which is defined as the short-axis radius (superior-inferior) divided by the long-axis radius (medial-lateral). Diaphragms that are more curved (e.g. normal fully-expired lung) will have a higher value index relative to more flattened (COPD fully-inspired lung). (b) Right (top) and left (bottom) examples of curvature indices over time. (c) Lung diaphragmatic distance (measured from lung apex to top of diaphragm) vs curvature index for the right (top) and left (bottom) diaphragms. (d) Example images during expiration (top) and inspiration (bottom) for subject data shown in B-C. (e) Lung apex to diaphragm distance vs curvature index for three pulmonary classes for largest breaths taken (defined by greatest lung area). Abbreviation: COPD, chronic obstructive pulmonary disease.

**e-Figure 6. Total lung areas also correlate with pulmonary function measures.** If suboptimal DDR tracking occurs, it usually involves the cardiac silhouette, thus an alternative is to track the entire lung area, without subtracting our heart area. (a) Example of total lung area tracking without removing heart silhouette. (b) Total lung capacity (PFT) vs maximum lung area (DDR). (c) Vital capacity (PFT) vs maximum-from-minimum lung area difference (DDR). (d) Forced expiratory volume in first second (PFT) vs difference of maximum positive and negative flow (DDR). (e) Inspiratory capacity (PFT) vs difference of maximum lung area from median minimum value (DDR). (f) Residual volume (PFT) vs minimum lung area (DDR). Pearson’s correlation r and significant (p) values shown. Abbreviations: DDR, dynamic digital radiography; FEV1, forced expiratory volume in one second; IC, inspiratory capacity; PFT, pulmonary function test; RV, residual volume; TLC, total lung capacity; VC, vital capacity.

**Dynamic Digital Radiography Pulmonary Function Testing: A Machine Learning Lung Study Alternative**

**Supplementary Table Legends**

**e-Table 1.** Number of patients per group that were included or excluded. Abbreviation: COPD, chronic obstructive pulmonary disease.

**e-Table 2.** Anatomical points included in each structure. Abbreviations: AArch, aortic arch; AK, aortic knob; Ao, aorta; At, atria; Ap, appendage; Cen, center; CW, chest wall; D, diaphragm; L, left; L#, left rib # (e.g L7, left rib 7); Lat, lateral; LU, left upper; Med, medial; PT, pulmonary trunk; R, right; R#, right ribs # (e.g. R7, right rib 7); RU, right upper; S, sternum.

**e-Table 3**. Strain groups and point-pairs they contain. Abbreviations: AArch, aortic arch; AK, aortic knob; Ao, aorta; At, atria; Ap, appendage; Cen, center; CW, chest wall; D, diaphragm; L, left; L#, left rib # (e.g L7, left rib 7); Lat, lateral; LU, left upper; Med, medial; PT, pulmonary trunk; R, right; R#, right ribs # (e.g. R7, right rib 7); RU, right upper; “_w_” denotes with.
